# The impact of area-based initiatives on physical activity trends in deprived areas; a quasi-experimental evaluation of the Dutch District Approach

**DOI:** 10.1186/1479-5868-11-36

**Published:** 2014-03-11

**Authors:** Daniëlle Kramer, Mariël Droomers, Birthe Jongeneel-Grimen, Marleen Wingen, Karien Stronks, Anton E Kunst

**Affiliations:** 1 Department of Public Health, Academic Medical Centre, University of Amsterdam, PO Box 22660, Amsterdam, 1100 DD The Netherlands; 2 Department of Social and Spatial Statistics, Statistics Netherlands, PO Box 4481, 6401 CZ Heerlen, The Netherlands

**Keywords:** Area-based initiatives, Evaluation, Physical activity, Quasi-experimental design, Deprivation

## Abstract

**Background:**

Numerous area-based initiatives (ABIs) have been implemented in deprived neighbourhoods across Europe. These large-scale initiatives aim to tackle the socio-economic and environmental problems in these areas that might influence physical activity (PA). There is little robust evidence of their impact on PA. This study aimed to assess the impact of a Dutch ABI called the District Approach on trends in leisure-time PA in deprived districts.

**Methods:**

Repeated cross-sectional data on 48401 adults across the Netherlands were obtained from the Integrated Survey on Household Living Conditions (POLS) 2004–2011. 1517 of these adults resided in deprived target districts and 46884 adults resided elsewhere in the Netherlands. In a quasi-experimental interrupted time-series design, multilevel logistic regression analyses were performed to assess trends in leisure-time walking, cycling, and sports before and during the intervention. Trends in deprived target districts were compared with trends in various control groups. The role of the intensity of environmental interventions was also assessed.

**Results:**

Deprived target districts showed a significantly positive change in walking trend between the pre-intervention and intervention period. The trend change in the deprived target districts was significantly larger compared to the rest of the Netherlands, but not compared to other deprived districts. For cycling and sports, neither deprived districts nor control districts showed a significant trend change. For all leisure-time PA outcomes, trend changes were not related to the intensity of environmental interventions in the deprived target districts.

**Conclusion:**

Some evidence was found to suggest that ABIs like the District Approach have a positive impact on leisure-time PA in deprived districts, regardless of the intensity of environmental interventions.

## Introduction

Residents of deprived neighbourhoods have consistently been found to be less physically active than residents of non-deprived neighbourhoods, independent of their individual socio-economic status [1-6]. In the past decade, numerous area-based initiatives (ABIs) have been implemented in deprived neighbourhoods across Western-Europe [7]. These large-scale initiatives aim to tackle the multitude of socio-economic and environmental problems in these neighbourhoods that might influence physical activity (PA) behaviour, including employment, income, housing, crime, and social cohesion. There is little robust evidence of their effect on PA [8-10]. Quasi-experimental evaluations of natural experiments may be useful to assess their effectiveness [10-13]. However, in the field of PA, this type of evaluations is still in its infancy [14].

ABIs may affect PA behaviour via different pathways. Better economic position may improve access to social and material resources for PA [15]. Stronger community bonds may enlarge social support and companionship for PA, which have consistently been associated with higher levels of PA [15,16]. Stronger community bonds may also reinforce positive social norms for healthy behaviours such as PA [15]. Improvements with respect to housing and the physical environment may improve neighbourhood aesthetics, pedestrian infrastructure and recreational facilities, which have all been consistently associated with PA [16-22]. A safer neighbourhood may reduce the fear of outdoor activities, although evidence for an association between neighbourhood safety and PA is less consistent [23].

The English New Deal for Communities (NDC) is one of the few ABIs that has been used as a natural experiment to explore its impact on PA [24,25]. The NDC aimed to improve the socio-economic and environmental situation in England’s most deprived areas. At 4-year and 6-year follow-up, a flat post-intervention trend in PA was found for NDC areas and control areas [24,25]. The authors concluded that the NDC had no impact on PA.

However, the impact of the NDC on PA may have been underestimated, as no pre-intervention trends were included in the evaluations. Moreover, previous research has suggested that the impact might depend on the ability of interventions to influence the outcome and on the quality of their implementation [9,26]. Interventions that are aimed at environmental problems instead of socio-economic problems may be more likely to cause district-wide changes in LTPA within a relatively short period of time because of a wider reach and shorter lag-times of effect. Studies are needed that assess changes in PA trends before and during the intervention and that focus on areas where intensive environmental interventions have been implemented.

An opportunity for such a study arose in 2008 with the implementation of a Dutch ABI called the District Approach. The District Approach aims to alleviate problems of employment, education, housing and the physical environment, safety, and social integration in 40 of the most deprived districts of the Netherlands. Districts have been selected based on their accumulation of economic, physical, and social problems, judged on statistics and survey data. Each district developed its own mix of socio-economic and environmental interventions, resulting in large between-district variations in the intensity of interventions [27].

This study aimed to evaluate the short-term impact of the District Approach on trends in leisure-time PA (walking, cycling, and sports) using a quasi-experimental interrupted time-series design. First, PA trends before and during the intervention were assessed in all deprived target districts and in various control groups. Next, trends were assessed in those deprived target districts where environmental interventions have been implemented most intensively. We expected to find a more positive PA trend change in deprived target districts than in the control groups, especially in deprived target districts with intensive environmental interventions.

## Methods

### Study population

Repeated cross-sectional data for years 2004 to 2011 were obtained from the Dutch Integrated Survey on Household Living Conditions (POLS). A random nationwide sample of non-institutionalized individuals of all ages was drawn from a subset of the national population registry. The subset consisted of individuals whose seventh digit of their personal identification number corresponded with the current year, e.g. 9 for 2009. This prevented individuals from being sampled twice over the years. Throughout the year, the individuals in the sample were approached by an interviewer for the basic survey. Questions on PA were asked in a supplementary internet or paper-and-pencil survey, administered only among individuals of age 12 and older. Between 2004 and 2011, 53778 individuals completed the additional survey. Total non-response was 40%.

For the analyses, respondents were excluded if younger than 18 and if PA scores were missing or unrealistic (scores exceeding 3360 minutes per week). Three different samples resulted as the number of missing and unrealistic scores was different for each of the PA outcomes. For walking, cycling, and sports, there were 48401, 48420, and 48906 adults with valid scores, respectively. Of these adults, 1517, 1544, and 1555, respectively, resided in deprived target districts. The remaining 46884, 46876, and 47351 adults, respectively, resided elsewhere in the Netherlands.

### Measures

#### Leisure-time physical activity

The dependent variable was self-reported leisure-time PA. PA was measured in POLS using the Dutch Short QUestionnaire to ASsess Health-enhancing physical activity (SQUASH). This instrument has shown to be fairly reliable and valid for measuring PA [28,29]. Respondents were asked to report the duration (hours and minutes per day) and frequency (days per week) of leisure-time walking, cycling, and sports. Water-related sports (e.g. skiing, surfing, diving) were excluded from the analyses because they were strongly bound to spaces that were usually outside of residential areas. Agility sports (e.g. bowling, darts, golf) and mental sports (e.g. chess) were excluded from the analyses because their intensity was too low to be considered as intense PA. Total minutes per week spent on leisure-time walking, cycling, and sports were calculated by multiplying duration and frequency. Distribution of the three PA outcomes was highly skewed, with almost half of the respondents not engaging in PA. Therefore, PA was dichotomised into ‘inactive’ (0 minutes per week) versus ‘active’ (any minutes per week). Sensitivity analyses have found results to be robust against alternative cut-off points (30 or 60 minutes per week) (results not shown).

#### Time variables

The main predictor variable was survey year. The survey was administered from 2004 to 2011. Respondents filled in the questionnaire at any time during these years. To make more accurate trend estimates, survey years were grouped into half year sections. One of the main effect modifiers was the survey period. Survey years were grouped into a pre-intervention period (January 2004 to June 2008) and an intervention period (July 2008 to December 2011).

#### Districts

The second main effect-modifier was the respondents’ district of residence. Data on 4-digit zip codes were obtained from the national population registry and linked to the POLS data. Subsequently, various groups of districts were identified. The main intervention group consisted of all deprived districts targeted by the District Approach. Three control groups were included. The first consisted of all other districts in the Netherlands (‘rest of the Netherlands’). As these districts may have been dissimilar at baseline in ways related to the study outcome, two additional control groups were included that were matched with the deprived target districts in terms of deprivation level and/or geographical location. The first matched control group consisted of other deprived districts with deprivation levels similar to that of the deprived target districts, but where the District Approach had not been introduced (‘other deprived districts’). The second matched control group consisted of only those other deprived districts that were located in the same city as the deprived target districts (‘other deprived districts same city’). Because of lower levels of power and the possibility of spill-over effects in the two matched control groups, the rest of the Netherlands was the main control group.

#### Intensity of environmental interventions

Within the District Approach, twelve types of environmental interventions were identified (housing quality, neighbourhood regeneration, green space, footpaths and cycle tracks, play grounds, sports facilities and activities, social capital, nuisance and conflicts, nuisance from youth, physical disorder, burglary, traffic safety). For each district, we first listed all activities implemented in each of these twelve fields of action for longer than one year [27]. Next, we specified the number of residents reached or the amount of neighbourhood change for each of the activities. Finally, this information was used to estimate the potential impact of all activities within a specific field of action. This impact was graded as low (no change expected in this field), intermediate (small changes expected) or high (substantial changes expected). For each district, we calculated overall intensity score by summing the grades in all twelve fields of action (low = 0, intermediate = 1, high = 2). The average overall intensity score over all areas was 11.47. We distinguished deprived target districts with less intensive environmental interventions (score ≤12, *n* = 16) from those with more intense environmental interventions (score ≥12, *n* = 20). For four districts, no detailed programme information was available. See Droomers et al. [27] for more detailed information on the implementation of interventions.

#### Potential confounders

Data on age (continuous), gender, household composition (five categories), and highest level of education completed (five ordinal groups based on the International Standard Classification of Education (ISCED)) were obtained from the POLS survey. Data on ethnicity (four categories) were derived from the national population registry. Information on equivalent disposable household income (quintiles) was obtained from the national tax registry.

### Statistical analyses

Interrupted time-series analyses were performed in 2013 to assess whether trends in leisure-time walking, cycling and sports have changed with the implementation of the District Approach. Multilevel logistic regression models were used to assess the association between year and PA, i.e. the half-yearly amount of change in prevalence of PA. Hereafter, this is called the *trend*. The trend was estimated for both the pre-intervention and intervention period. The difference in trends between these two periods was assessed by means of an interaction term for year and period. Hereafter, this will be called the *trend change*.

An interaction term for year and district was included to assess differences in PA trends between the deprived target districts and the various control groups. An interaction term for year, district, and period was included to assess differences in PA trend changes between the deprived target districts and the various control groups. The role of the intensity of the environmental interventions was assessed by focussing the analyses on deprived target districts with either less intensive or more intensive environmental interventions.

Analyses were adjusted stepwise. Model 1 controlled for age, gender, household composition, and ethnicity. To explore the moderating effect of socio-economic factors, model 2 further controlled for education and income. Multilevel regression analyses were applied to take clustering of respondents in districts into account. Level 1 represented individuals and level 2 represented neighbourhoods. All analyses were carried out using STATA 11.0 software. Statistical significance was set at 0.05.

This study was based on secondary analyses of anonymized survey data. The Medical Ethics Committee of the Academic Medical Centre in Amsterdam, the Netherlands, has confirmed that ethics approval is not necessary, because the Medical Research Involving Human Subjects Act (WMO) does not apply to our study.

## Results

Residents of the deprived target districts differed significantly from those of the control groups, especially from the rest of the Netherlands, with respect to most characteristics (Table 1). Compared with all control groups, a higher percentage of residents of deprived target districts was single, non-Western non-ethnic Dutch, had lower educational levels, had lower income levels, and did no leisure-time cycling and sports. No between-district differences were noted in the average prevalence of leisure-time walking.

**Table 1:** Characteristics of the study population

	**Total**	**Deprived target districts**	**Control groups**		
			**Rest of the Netherlands**	**Other deprived districts**^**b**^	**Other deprived districts, same city**^**c**^
**Numbers**					
*n* 4-digit zipcodes	3502	83	3419	250	119
*n* adults in total	48401	1517	46884	4277	2389
*n* adults per half year (mean ± SD)	3025 ± 293	95 ± 16	2 930 ± 286	267 ± 26	149 ± 15
*n* adults per zipcode (mean ± SD)	14 ± 12	18 ± 8	14 ± 12	17 ± 11	20 ± 11
**Characteristics**^**a**^					
**Age (mean ± SD)**	49.4 ± 16.9	48.1 + 17.9	49.4 + 16.9*	48.8 + 17.2	48.2 + 17.0
**Gender (%)**					
Men	47.7	46.1	47.8	46.0	45.6
Women	52.3	53.9	52.2	54.0	54.4
**Household composition (%)**			*	*	*
Partner/married with child (ren)	39.4	35.2	39.5	33.9	33.2
Partner/married without child (ren)	38.4	28.3	38.7	36.1	32.7
Single without child (ren)	16.9	25.4	16.6	23.0	26.2
Single with child (ren)	4.1	8.2	4.0	5.1	5.6
Other	1.2	2.8	1.1	1.9	2.3
**Ethnicity (%)**			*	*	*
Ethnic Dutch	87.8	66.1	88.5	80.5	77.2
Non-ethnic Dutch, western	7.1	9.7	7.1	9.4	10.6
Non-ethnic Dutch, non-western	3.4	19.1	2.8	7.1	8.8
Non-ethnic Dutch, origin unknown	1.2	3.3	1.1	1.8	2.2
**Education (%)**			*	*	*
Primary education	13.4	22.0	13.2	16.9	15.9
Secondary education: lower level	23.3	25.1	23.2	20.6	17.6
Secondary education: higher level	35.1	29.1	35.3	29.9	26.8
Tertiary education	26.3	21.9	26.4	30.3	37.0
**Income (%)**			*	*	*
First quintile (< €15037)	16.5	27.9	16.1	21.3	22.6
Second quintile (€15037 - €19000)	18.9	23.8	18.7	20.2	18.9
Third quintile (€19001 - €23317)	19.8	19.6	19.8	19.0	17.8
Fourth quintile (€23318 - €29746)	21.3	15.0	21.5	19.0	18.5
Fifth quintile (> €29746)	22.0	11.7	22.3	19.0	20.7
**Physical activity (% active)**					
Leisure-time walking	62.6	63.3	62.6	60.8	62.7
Leisure-time cycling	54.6	42.0	55.0*	49.5*	48.7*
Sports	43.0	36.7	43.2*	41.2*	42.9*

*Differs significantly from deprived target districts.

^a^Percentages may not add up to 100% due to the category ‘missings’ which has not been reported.

^b^Districts with levels of deprivation similar to that of the deprived target districts, but where the District Approach had not been introduced.

^c^Districts with levels of deprivation similar to that of the deprived target districts and that are situated in the same cities as the deprived target districts, but where the District Approach had not been introduced.

**Figure 1:**
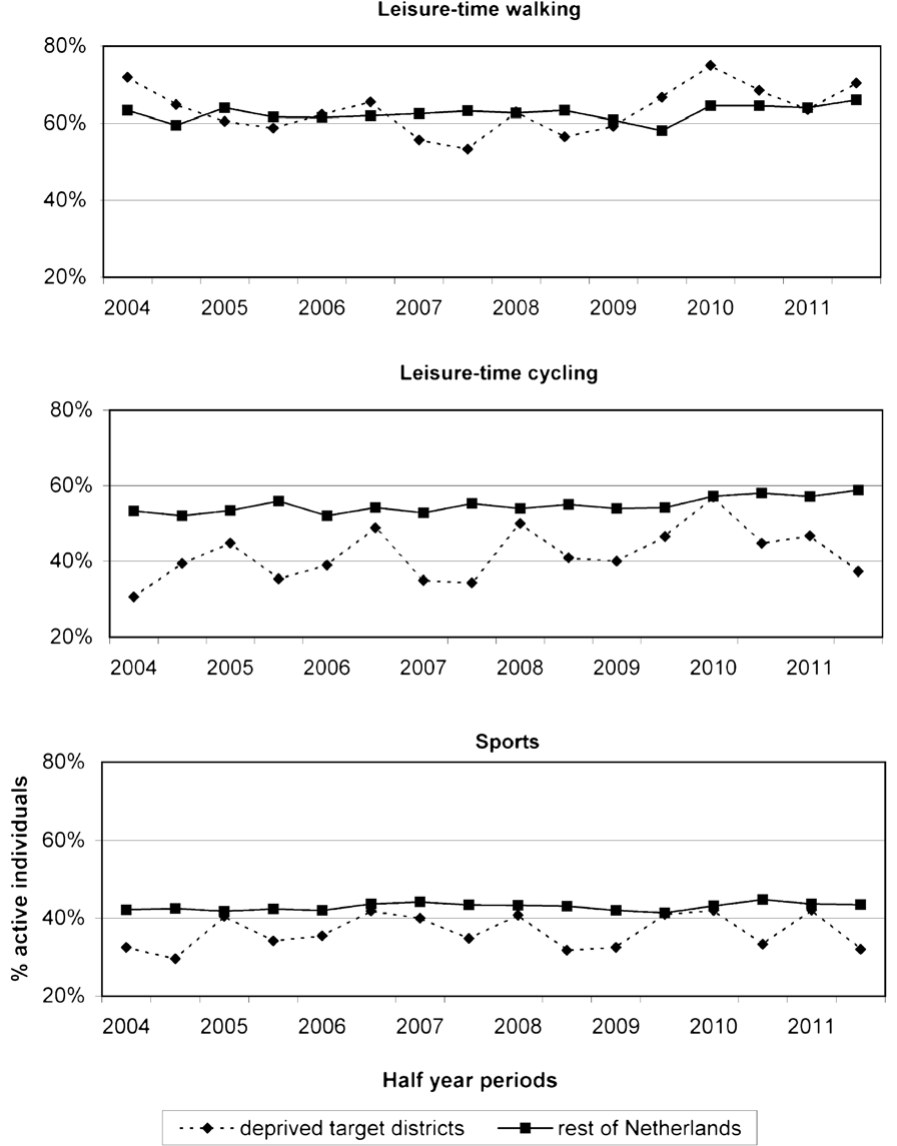
Trends in leisure-time physical activity in deprived target districts and the rest of the Netherlands.

Figure 1 displays the trends in prevalence of leisure-time walking, cycling, and sports between 2004 and 2011. In the deprived target districts, prevalence of cycling slightly increased between 2004 and 2011, while prevalence of sports remained unchanged. For walking, the trend in deprived target districts differed between the pre-intervention and intervention period. In the pre-intervention period, prevalence decreased from 72% in the first half of 2004 to 63% in the first half of 2008. In the intervention period, prevalence increased from 57% in the second half of 2008 to 70% in the second half of 2011. For all PA outcomes, the rest of the Netherlands showed a steady trend over the years.

Table 2 shows the change in PA trends between the pre-intervention period and the intervention period for deprived target districts and the rest of the Netherlands. In deprived target districts, the trend in walking changed from a slightly negative trend before the District Approach (β: −0.04; 95% confidence interval (CI): −0.08 – 0.00) to a positive trend during the District Approach (β: 0.11; 95% CI: 0.04 – 0.18). This change in trend was found to be significant (β: 0.15; 95% CI: 0.04 – 0.25). In the rest of the Netherlands too, there was a significantly positive trend change in walking, though smaller (β: 0.03; 95% CI: 0.01 – 0.04). The trend change in walking was significantly more positive in the deprived target districts than in the rest of the Netherlands (β: 0.12; 95% CI: 0.02 – 0.22). Adjustment for potential confounders did not substantially alter the results. For cycling and sports, trend changes were slightly negative in deprived target districts. However, trend changes were not significant and did not significantly differ from those in the rest of the Netherlands.

**Table 2:** **Trends in leisure**-**time physical activity in deprived target districts versus the rest of the Netherlands**

		**Trend in walking/cycling/sports**^**a **^**(regression coefficient β (95% confidence interval))**		
**Model**^**b**^	**Period**	**Pre intervention**	**Intervention**	**Intervention versus pre intervention**
	**District type**			
**Leisure-time walking**				
M0	Deprived target districts	−0.04 (−0.08 – 0.00)	0.11 (0.04 – 0.18)*	0.15 (0.04 – 0.25)*
	Rest of the Netherlands	−0.00 (−0.01 – 0.00)	0.02 (0.01 – 0.04)*	0.03 (0.01 – 0.04)*
	Deprived target districts versus rest of the Netherlands			0.12 (0.02 – 0.22)*
M1	Deprived target districts	−0.04 (−0.08 – 0.01)	0.11 (0.04 – 0.19)*	0.15 (0.04 – 0.30)*
	Rest of the Netherlands	−0.00 (−0.01 – 0.00)	0.02 (0.01 – 0.03)*	0.03 (0.01 – 0.04)*
	Deprived target districts versus rest of the Netherlands			0.12 (0.02 – 0.23)*
M2	Deprived target districts	−0.04 (−0.08 – 0.00)	0.11 (0.03 – 0.18)*	0.14 (0.04 – 0.25)*
	Rest of the Netherlands	−0.00 (−0.01 – 0.00)	0.02 (0.01 – 0.03)*	0.02 (0.00 – 0.04)*
	Deprived target districts versus rest of the Netherlands			0.12 (0.02 – 0.23)*
**Leisure-time cycling**				
M0	Deprived target districts	0.04 (0.00 – 0.08)*	0.00 (−0.07 – 0.07)	−0.04 (−0.14 – 0.06)
	Rest of the Netherlands	0.00 (−0.00 – 0.01)	0.03 (0.02 – 0.04)*	0.02 (0.01 – 0.04)*
	Deprived target districts versus rest of the Netherlands			−0.06 (−0.16 – 0.04)
M1	Deprived target districts	0.05 (0.00 – 0.09)*	0.00 (−0.07 – 0.07)	−0.04 (−0.14 – 0.06)
	Rest of the Netherlands	0.01 (−0.00 – 0.01)	0.03 (0.02 – 0.04)*	0.03 (0.01 – 0.04)*
	Deprived target districts versus rest of the Netherlands			−0.07 (−0.17 – 0.03)
M2	Deprived target districts	0.05 (0.01 – 0.09)*	−0.00 (−0.07 – 0.07)	−0.05 (−0.15 – 0.05)
	Rest of the Netherlands	0.01 (−0.00 – 0.01)	0.03 (0.01 – 0.04)*	0.02 (0.00 – 0.04)*
	Deprived target districts versus rest of the Netherlands			−0.07 (−0.17 – 0.03)
**Sports**				
M0	Deprived target districts	0.02 (−0.02 – 0.06)	−0.01 (−0.08 – 0.06)	−0.03 (−0.13 – 0.07)
	Rest of the Netherlands	0.00 (−0.00 – 0.01)	0.00 (−0.01 – 0.02)	0.00 (−0.02 – 0.02)
	Deprived target districts versus rest of the Netherlands			−0.03 (−0.13 – 0.07)
M1	Deprived target districts	0.02 (−0.02 – 0.06)	−0.02 (−0.09 – 0.05)	−0.04 (−0.14 – 0.07)
	Rest of the Netherlands	0.01 (0.00 – 0.02)*	0.01 (0.00 – 0.02)*	0.00 (−0.02 – 0.02)
	Deprived target districts versus rest of the Netherlands			−0.04 (−0.14 – 0.07)
M2	Deprived target districts	0.02 (−0.02 – 0.06)	−0.04 (−0.12 – 0.03)	−0.06 (−0.17 – 0.04)
	Rest of the Netherlands	0.00 (−0.00 – 0.01)	−0.01 (−0.02 – 0.00)	−0.01 (−0.03 – 0.01)
	Deprived target districts versus rest of the Netherlands			−0.05 (−0.16 – 0.06)

*P ≤ 0.05.

^a^Trend represents the half yearly change in ln (odds).

^b^M0: unadjusted. M1: adjusted for age, gender, household composition, ethnicity. M2: additional adjustment for education, income.

For walking, differences in trend change were of similar magnitude but not statistically significant when deprived target districts were compared with two matched control groups (Table 3). The trend change was slightly more positive in the two matched control groups than in the rest of the Netherlands. As a result, the significantly positive trend change in deprived target districts did not significantly differ from that in the two matched control groups. For cycling and sports, trend changes were similar across all control groups.

**Table 3:** **Trends in leisure**-**time physical activity in deprived target districts versus various control groups**

	**Trend in walking/cycling/sports**^**a **^**(regression coefficient β (95% confidence interval))**		
**Period**	**Pre intervention**	**Intervention**	**Intervention versus pre intervention**
	**District type**			
**Leisure-time walking**				
Deprived target districts	−0.04 (−0.08 – 0.00)	0.11 (0.03 – 0.18)*	0.14 (0.04 – 0.25)*	
Rest of the Netherlands	−0.00 (−0.01 – 0.00)	0.02 (0.01 – 0.03)*	0.02 (0.00 – 0.04)*	
Deprived target districts versus rest of the Netherlands			0.12 (0.02 – 0.23)*	
Other deprived districts	−0.01 (−0.04 – 0.01)	0.02 (−0.02 – 0.06)	0.04 (−0.02 – 0.10)	
Deprived target districts versus other deprived districts			0.11 (−0.01 – 0.23)	
Other deprived districts, same city	−0.02 (−0.05 – 0.01)	0.04 (−0.01 – 0.10)	0.06 (−0.02 – 0.14)	
Deprived target districts versus other deprived districts, same city			0.09 (−0.04 – 0.22)	
**Leisure-time cycling**				
Deprived target districts	0.05 (0.01 – 0.09)*	−0.00 (−0.07 – 0.07)	−0.05 (−0.15 – 0.05)	
Rest of the Netherlands	0.01 (−0.00 – 0.01)	0.03 (0.01 – 0.04)*	0.02 (0.00 – 0.04)*	
Deprived target districts versus rest of the Netherlands			−0.07 (−0.17 – 0.03)	
Other deprived districts	0.01 (−0.02 – 0.03)	0.05 (0.01 – 0.09)*	0.04 (−0.02 – 0.10)	
Deprived target districts versus other deprived districts			−0.10 (−0.22 – 0.02)	
Other deprived districts, same city	0.01 (−0.02 – 0.05)	0.03 (−0.02 – 0.08)	0.02 (−0.06 – 0.09)	
Deprived target districts versus other deprived districts, same city			−0.08 (−0.21 – 0.04)	
**Sports**				
Deprived target districts	0.02 (−0.02 – 0.06)	−0.04 (−0.12 – 0.03)	−0.06 (−0.17 – 0.04)	
Rest of the Netherlands	0.00 (−0.00 – 0.01)	−0.01 (−0.02 – 0.00)	−0.01 (−0.03 – 0.01)	
Deprived target districts versus rest of the Netherlands			−0.05 (−0.16 – 0.06)	
Other deprived districts	0.00 (−0.02 – 0.03)	0.01 (−0.04 – 0.05)	0.00 (−0.06 – 0.07)	
Deprived target districts versus other deprived districts			−0.05 (−0.18 – 0.07)	
Other deprived districts, same city	−0.01 (−0.04 – 0.03)	−0.01 (−0.07 – 0.04)	−0.00 (−0.09 – 0.08)	
Deprived target districts versus other deprived districts, same city			−0.05 (−0.18 – 0.08)	

*P ≤ 0.05.

^a^Trend represents the half yearly change in ln (odds), adjusted for age, gender, household composition, ethnicity, education, and income.

Table 4 shows the change in PA trends for deprived target districts with less and more intensive environmental interventions. Both low- and high-intensity districts showed a positive trend change in walking. The trend change was somewhat larger in low-intensity districts (β: 0.20; 95% CI: 0.01 – 0.40) than in high-intensity districts (β: 0.10; 95% CI: −0.04 – 0.24), but confidence intervals greatly overlapped. For both districts, trend changes did not significantly differ from those in the rest of the Netherlands. For cycling, trend changes were similar in the high- and low-intensity districts. Trend changes in both districts were slightly more negative than in the rest of the Netherlands, but differences were small and not statistically significant. For sports, no trend change was apparent in low-intensity districts (β: −0.01; 95% CI: −0.21 – 0.19), while high-intensity districts showed a slightly negative trend change (β: −0.12; 95% CI: −0.26 – 0.02). Again, confidence intervals greatly overlapped. The trend change in high-intensity districts was not significant and did not significantly differ from that in the rest of the Netherlands (β: −0.11; 95% CI: −0.25 – 0.03).

**Table 4:** **Trends in leisure**-**time physical activity in deprived target districts with less and more intensive environmental interventions**

	**Trend in walking/cycling/sports**^**a **^**(regression coefficient β (95% confidence interval))**		
**Period**	**Pre intervention**	**Intervention**	**Intervention versus pre intervention**
	**District type**			
**Leisure-time walking**				
Rest of the Netherlands	−0.00 (−0.01 – 0.00)	0.02 (0.01 – 0.03)*	0.02 (0.00 – 0.04)*	
Low-intensity deprived target districts	−0.07 (−0.15 – 0.01)	0.13 (−0.00 – 0.27)	0.20 (0.01 – 0.40)*	
Low-intensity deprived target districts versus rest of the Netherlands			0.18 (−0.01 – 0.38)	
High-intensity deprived target districts	−0.02 (−0.07 – 0.04)	0.08 (−0.01 – 0.18)	0.10 (−0.04 – 0.24)	
High-intensity deprived target districts versus rest of the Netherlands			0.08 (−0.06 – 0.22)	
**Leisure-time cycling**				
Rest of the Netherlands	0.01 (−0.00 – 0.01)	0.03 (0.01 – 0.04)*	0.02 (0.00 – 0.04)*	
Low-intensity deprived target districts	0.04 (−0.04 – 0.11)	−0.03 (−0.16 – 0.11)	−0.06 (−0.25 – 0.13)	
Low-intensity deprived target districts versus rest of the Netherlands			−0.08 (−0.27 – 0.11)	
High-intensity deprived target districts	0.06 (−0.00 – 0.11)	0.01 (−0.08 – 0.10)	−0.05 (−0.18 – 0.08)	
High-intensity deprived target districts versus rest of the Netherlands			−0.07 (−0.20 – 0.06)	
**Sports**				
Rest of the Netherlands	0.01 (−0.00 – 0.01)	−0.01 (−0.02 – 0.00)	−0.01 (−0.03 – 0.01)	
Low-intensity deprived target districts	−0.00 (−0.09 – 0.08)	−0.02 (−0.15 – 0.12)	−0.01 (−0.21 – 0.19)	
Low-intensity deprived target districts versus rest of the Netherlands			−0.00 (−0.20 – 0.20)	
High-intensity deprived target districts	0.04 (−0.02 – 0.10)	−0.08 (−0.17 – 0.02)	−0.12 (−0.26 – 0.02)	
High-intensity deprived target districts versus rest of the Netherlands			−0.11 (−0.25 – 0.03)	

*P ≤ 0.05.

^a^Trend represents the half yearly change in ln (odds), adjusted for age, gender, household composition, ethnicity, education, and income.

## Discussion

This study provides novel insights into the impact of ABIs on leisure-time PA. In the deprived target districts, there was a positive trend change in walking between the periods before and during the District Approach. This trend change was significantly larger than in the rest of the Netherlands. Neither deprived target districts nor control districts showed a significant trend change in cycling or sports. Trend changes in PA appeared to be unrelated to the intensity of environmental interventions in the deprived target districts.

### Limitations

Total non-response was 40%. Non-response may have been selective in ways related to our study outcome. A comparison of weighed and non-weighed characteristics of the total study population revealed a small overrepresentation of older people, women, couples with children, ethnic Dutch, people with higher income, and active people (data not shown). Unfortunately, information on non-response was not available for the deprived target districts specifically. However, even if non-response would have been selective in the deprived target districts, it would have only affected our trend estimates if non-response rates would have differed over time. In our study, non-response rates appeared to remain stable over time.

We used repeated cross-sectional data for time points of half year each. The characteristics of the survey samples may have varied between these half years. However, given the sampling design that was used, there is little reason to expect the time variation to be systematic. Moreover, we controlled our analyses for possible systematic variations in the demographic and socio-economic characteristics of respondents.

We compared trends in deprived target districts with those in various control groups. Comparison with other deprived districts has the advantage of increasing similarity between the intervention and control groups. On the downside, these districts may have received ABIs similar to the District Approach. Moreover, deprived control districts that are located near the deprived target districts might have experienced spill-over effects of the District Approach. Comparison with such districts may thus cause an underestimation of the impact of the District Approach. The use of a national control group minimized these problems, at the price of greater dissimilarity between the intervention and control group. To control for some of these dissimilarities in population composition, analyses were adjusted for various demographic and socio-economic characteristics.

Our results might have been biased by selective migration of residents [11,30]. The District Approach might have led to the migration of more affluent (and active) individuals into the deprived target districts. Consequently, changes in PA might have been the result of population changes rather than environmental changes. However, such selective migration seems to have played a minor role as adjustment for socio-economic factors did not substantially change our key findings. Moreover, previous evaluation studies of the NDC have found changes in PA to be similar in high- and low-mobility areas, indicating selective migration to be absent [30].

This evaluation study has a limited post-intervention evaluation time of 3.5 years. Longer follow-up time is needed to address the long-term impact of the District Approach on PA.

### Interpretation of results

Evaluation studies of the NDC found no evidence of an effect on PA [24,25]. We initially hypothesised this absence of effect to be partly due to lack of inclusion of the pre-intervention trend. In the current study, however, key results were similar regardless of the inclusion of pre-intervention trends.

The NDC evaluations examined overall PA [24,25]. Results of the current study illustrate the need to distinguish different types of PA, as a positive effect of the District Approach was observed for leisure-time walking only, and not for leisure-time cycling or sports. The District Approach might have had an impact on leisure-time walking only, as walkers are most likely to be exposed to their immediate neighbourhood environment. In the Netherlands, 40% of the walking trips take place within 5 kilometres of home, compared to only 4% of the cycling trips [31]. Exposure to the neighbourhood environment is limited with many sports, as more than half of the sports activities take place at indoor sports clubs [32]. A recent review has also found various aspects of the physical neighbourhood environment to be associated with walking, but less so with other types of PA [33].

Trends in walking in deprived target districts were not related to the intensity of environmental interventions. Perhaps PA trends were affected less by environmental interventions and more by interventions that were aimed at the individual, such as employment- and education-related initiatives. However, we found no indications for an impact of individual-level interventions on PA. First, adjustment for education and income levels had little or no effect on the results. Second, individual-level interventions as carried out in the District Approach reached only a small part of the population, and their impact on PA among these people is likely to have long lag-times. As a result, it is unlikely that the individual-level interventions have caused short-term district-wide changes in PA.

The finding that PA trends were unrelated to the intensity of interventions suggests that factors other than intensity were more important determinants of the outcomes of the District Approach. Prior research of the NDC has also found expenditure and number of interventions to be unrelated to the outcome of the programme [34]. The impact of the NDC on various place- and people-based outcomes was found to vary according to the size and socio-demographic composition of the population, the urbanization level of the area, and the amount of problems in the area at the start of the programme [34]. Unfortunately, we were unable to make such distinctions.

Future studies should use longitudinal data to prevent variations in study population over the years, which may result either from selective migration or from variations in sampling. Longitudinal studies may also be useful to explore the underlying mechanisms of change, for example by relating PA changes to changes in socio-economic and environmental factors, or by comparing PA changes between beneficiaries and non-beneficiaries of specific projects. Moreover, future studies should take into account the local context to capture possible variations in effect between areas.

## Conclusions

There was some evidence that ABIs like the Dutch District Approach might have a positive effect on leisure-time PA behaviour in deprived areas. The District Approach appeared to improve trends in leisure-time walking, regardless of the intensity of environmental interventions. By applying a quasi-experimental time-series design, this study offers new evidence for the impact of ABIs on leisure-time PA. This design could be applied to future evaluations of ABIs. However, complementary studies are needed to uncover the mechanisms through which ABIs might affect leisure-time PA behaviour in deprived districts.

## Competing interests

The authors declare that they have no competing interests.

## Authors’ contributions

DK and AEK developed the study design. DK and MW prepared the data. DK performed the statistical analyses and MW checked the execution of the analyses. With the help of AEK, DK wrote a draft of the manuscript. AEK, MD, BJ-G, KS and MW critically reviewed intermediate results and manuscript versions and made substantial contributions to subsequent versions. All authors have read and approved the final version of the manuscript.
